# Development of a context-sensitive physical activity intervention for persons living with HIV and AIDS of low socioeconomic status using the behaviour change wheel

**DOI:** 10.1186/s12889-019-7091-8

**Published:** 2019-06-17

**Authors:** S.Z. Mabweazara, L.L. Leach, C. Ley

**Affiliations:** 1 Department of Sport, Recreation & Exercise Science, Faculty of Community and Health Sciences, University of the Western Cape, Cape Town, South Africa; 2 Institute of Sport Science, University of Vienna, Auf der Schmelz 6, 1150 Vienna, Austria; 3 Department of Health Sciences, University of Applied Sciences FH Campus Wien, Vienna, Austria

**Keywords:** HIV, AIDS, Physical activity, Behaviour change wheel, Socioeconomic status

## Abstract

**Background:**

Regular physical activity (PA) has been recommended for the management of HIV and AIDS. The purpose of this study was to develop a contextualised intervention for promoting PA among women living with HIV and AIDS (WLWHA) of low socioeconomic status (SES). A secondary aim of the study was to optimise the PA intervention using behavioural theory/ frameworks derived from preliminary studies and the literature.

**Methods:**

The Behaviour Change Wheel (BCW) for designing behaviour change interventions was used. This method was further supplemented by evidence from the literature, systematic literature review (SLR), a concurrent mixed methods study and two cross-sectional studies. The SLR aided in determining the theoretical frameworks to inform the intervention, the specific PA behaviours to be targeted by the intervention, the intervention functions, the intervention policy category and the mode of delivery of the intervention. The concurrent mixed methods study was used to identify key factors that needed to change in order for participants to engage in regular PA. The first cross-sectional study was used to determine the gender to be targeted by the study. The second cross-sectional study was used to determine the domain and intensity of PA to target in the intervention.

**Results:**

A face-to-face context-sensitive PA intervention employing 14 behavioural change techniques was designed. The PA intervention (a) utilised the Transtheoretical model of behaviour change and the Social Cognitive theory as the underpinning theoretical frameworks (b) included convenient PAs, such as walking, doing simple home-based exercises, engaging in activities of daily living or doing simple exercises at the community centre (c) used education, reward, training in PA, modelling exercise activities and enablement to increase the opportunity to engage in PA as intervention functions (d) used service provision as policy priorities, and (e) used a direct face-to-face mode of delivery.

**Conclusions:**

The PA intervention emphasises behavioural techniques for increasing PA participation, such as goal-setting, self-monitoring, strategies for overcoming PA barriers, social support and rewards. The intervention employs strategies that highlight low-cost local PA resources and opportunities to help HIV infected women of low SES to participate in PA. The BCW provides a useful and comprehensive framework for the development of evidence and theory-based PA interventions for PLWHA of low SES. The BCW can thus be used in the development of interventions that ‘talk’ to policy by bridging the health inequality gap.

## Background

Amongst the general population, physical activity (PA) has been found to have both psychological and physical health benefits [1, 2]. Regular PA has also been prescribed as an alternative disease management strategy for HIV and AIDS [3, 4]. A systematic review of literature showed that PA and exercise are safe and effective methods of enhancing cardiorespiratory fitness, metabolic function and quality of life among people living with HIV and AIDS (PLWHA) [5]. Similarly, progressive resistance exercise aids in improving body composition and muscular strength among PLWHA [6]. Long-term exercise training lowers the likelihood of disability caused by sarcopenia among PLWHA [7].

The use of highly active antiretroviral therapy (HAART) to manage the effects of HIV/AIDS was associated with adverse morphological conditions such as lipoatrophy (loss of fat in the facial area and the upper and lower limbs) and lipohypertrophy (fat accumulation in the abdominal, cervical and breast areas) [8]. PA has been found to mitigate the adverse effects of HAART [8]. Unfortunately, PLWHA of low SES, particularly women are at a greater risk of low PA [9, 10] and this is usually related to low SES [11]. Accordingly, Mabweazara, Leach & Ley [12] have advocated for the development of contextualised and theoretically informed PA interventions for PLWHA of low SES.

In spite of the evidence of the health benefits of PA, evidence suggests that PLWHA in Africa do not engage in adequate amounts of PA [10, 13–15]. A factor that has been found to influence the low PA levels amongst PLWHA in Africa and in Western societies is socioeconomic status (SES) [16, 17]. Likewise, in Africa, HIV and AIDS are diseases that predominantly affect persons of low SES [18, 19]. Therefore, in Africa, PLWHA are more likely to be of low SES, and are more likely to be inactive [20]. This is so because, most people living with HIV are found in low-and middle-income countries, with close to 66% located in sub-Saharan Africa [21]. In South Africa specifically, the number of people living with HIV increased from an estimated 4.25 million in 2002 to 7.52 million by 2018 [22]. Furthermore, self-reported data from 51 mostly low-and middle-income countries showed that one-fifth of adults are classified as physically inactive [23]. Consequently, health practitioners need to develop PA interventions that are applicable and match the African context.

However, for persons of low SES, care must be taken not to come to false conclusions, especially with regards to their PA levels when using survey questionnaires. Evidence from the literature might suggest that persons of low SES do not engage in adequate PA, but persons of low SES, for example, are bound to engage in transport-related PA as most do not own cars [24] and are also highly likely to engage in employment-related PA, as most are employed in active occupations [11]. Both transport-related PA and employment-related PA may not be construed as PA to them, as they also form part of their occupation-related activities and activities of daily living (ADLs). An important aspect of contextualising the research process, therefore, is to bring more into the focus to persons of low SES, that PA maybe embedded in ADLs. As such, survey questionnaires and interview questions for these individuals must be grounded on a contextualised definition of PA that also considers ADLs. A contextualised definition of PA for persons of low SES might be “Any bodily movements caused by skeletal muscles that are associated with an increase in energy demand that might be done through structured exercises, ADLs, work-related activities or walking from one place to another”.

One way of developing informed and successful interventions is the use of established methods for intervention design. As such, health practitioners should utilise a methodical approach in the intervention design process [25]. This entails using a systematised approach in the design of the intervention, such as an established design framework that will assist the health practitioner to contextualise and select the appropriate intervention ingredients that have the potential of bringing about the desired behaviour change. One such framework is the Behaviour Change Wheel (BCW) that provides a comprehensive, systematic and transparent approach to intervention design based on established behaviour change theory [26]. The BCW is specifically appropriate for promoting PA among persons of low SES because it also incorporates behaviour change techniques that are grounded in theories (e.g., Social cognitive theory [SCT] and the Transtheoretical model [TTM]) that have been found to be effective in promoting PA amongst persons of low SES [12].

In a systematic review for identifying effective theories and BCTs for informing PA interventions for low SES populations, Mabweazara et al. [12] suggest that South African researchers, specifically, should aim at developing contextualised PA interventions for the management of HIV and AIDS. Pharmacological therapies that may alleviate some of the detrimental effects of HIV are linked to exorbitant financial costs [27]. Therefore, the practise of simple low-cost PA should be encouraged in PLWHA of low SES [28]. Given the foregoing, the purpose of this study was to develop a contextualised intervention for promoting PA among persons living with HIV and AIDS (PLWHA) of low socioeconomic status (SES). A secondary aim of the study was to optimise the PA intervention using behavioural theory/ frameworks derived from preliminary studies and the literature.

## Methods

### Aim, design and setting

The purpose of this study was to develop a contextualised intervention for promoting PA among PLWHA of low SES. In addition to the BCW and the TDF [29], this study was based on a number of studies that were conducted to aid in the intervention design process. These included a systematic review [12], a concurrent mixed methods study [30], and two cross-sectional studies [11, 31]. However, identifying the intervention functions, policy categories and mode of delivery may require the use of judgement in order to make the most appropriate decisions in the context of a planned intervention [29]. As such, the assessment of affordability, practicability, effectiveness, acceptability, side effects and safety and equity referred to as the APEASE criteria (see Table 2) outlined in the BCW guide was used to make strategic judgements regarding intervention content and mode of delivery by the research team (SZM, LLL, CL). Table 2 shows the APEASE criteria for designing and evaluating interventions.

### Materials and processes

The preliminary studies conducted to aid in the intervention design process were as follows:

1. A systematic review was conducted to inform the design of PA interventions for low-income persons by identifying successful behavioural change techniques (BCTs) and theoretical frameworks [12]. The review included randomized controlled trials with interventions aiming to promote PA and/or adherence to PA for the management of chronic disease. The systematic review specifically focused on chronic disease, because HIV infection is now considered a chronic disease [32].

#### Findings

Eleven studies met the inclusion criteria. Amongst other behavioural change techniques (BCTs) reported in chapter 3, ‘provide feedback on performance’, ‘goal setting (behaviour)’, and ‘plan social support/social change’ were the most frequently used behavioural change techniques. Other BCTs that were identified as successful interventions were prompting self-monitoring of behavioural outcomes, providing information about where and when to perform the behaviour, using follow-up prompts, barrier identification/problem solving, prompt review of behavioural goals, prompt self-monitoring of behaviour, action planning, providing rewards contingent on successful behaviour, providing instruction on how to perform the behaviour, relapse prevention/coping planning, motivational interviewing, prompt reviewing of outcome goals, providing information on the consequences of behaviour to the individual, setting graded tasks, environmental structuring, model/demonstrate behaviour, providing information on the consequences of behaviour in general, facilitating social comparison, teaching use of prompts/cues and time management. Among the existing theories, the Transtheoretical model (TTM) and the Social cognitive theory (SCT) were the common theoretical frameworks to underpin most study interventions. A noteworthy observation of this review was that only one of the 11 studies focused on promoting PA among PLWHA (Webel, Moore, Hanson & Salata, 2013). Table 3 shows the outcome of extracting these intervention aspects from the 11 studies included in the systematic review.

#### Contributions of the study towards intervention development

BCTs identified through this review, as well as the BCW were implemented in the final intervention. Theoretical frameworks identified through this review were used to inform the intervention. The systematic review was also used to determine specific PA behaviours targeted by the intervention, the intervention functions, the intervention policy category and the mode of delivery.

2. A cross-sectional study with 978 HIV positive participants of low SES was conducted to determine if age, body weight, height, gender, waist-to-hip ratio (WHR), educational attainment, employment status, CD4+ cell count, and body mass index (BMI) could predict overall PA among PLWHA of low SES [31].

#### Findings

It was found that education, employment status and gender significantly predicted total moderate-to-vigorous PA (TMVPA) among PLWHA of low SES. Gender had the greatest effect on TMVPA compared to education and employment. Women engaged in low levels of PA compared to men.

#### Contributions of the study towards intervention development

The intervention targeted unemployed WLWHA with low education levels. Since education was a significant predictor of PA, the intervention included educating the participants about the health benefits of PA, the strategies for overcoming the barriers to PA participation, and the strategies of social support and self-efficacy to enhance PA participation. A PA promotion pamphlet with the relevant information and home-based exercise activities was also prepared as part of the intervention material. Thus, the PA intervention educated WLWHA on the benefits of PA for the management of HIV and AIDS and teaching them simple low-cost exercises they could do at home.

3. A cross-sectional study with 978 HIV positive participants of low SES was conducted to examine the PA profile of PLWHA based on PA domains and PA intensity. The study also sought to determine whether employment status and level of education could predict PA among PLWHA of low SES [11].

#### Findings

The findings of the study showed that PLWHA engaged most frequently in work-related PA, followed by transport-related PA and, lastly, in leisure-related PA. Participants engaged more frequently in overall PA at a moderate- than vigorous-intensity of PA. Employment was also a significant predictor of work-related PA. The results showed that being unemployed was related with lower levels of work-related PA.

#### Contributions of the study towards intervention development

The PA intervention targeted unemployed WLWHA. The PA intervention included strategies of dealing with barriers to PA in low-income settings. The PA intervention promoted walking as transport-related PA. There was emphasis on engaging in leisure-related PA. The intervention also emphasised on engaging in regular low-to moderate intensity PA. Personalised PA goals were set for each participant, because of the variations in ability, especially due to fluctuations in CD4+ cell counts.

4. A concurrent mixed method study was conducted with 21 HIV positive female participants of low SES using the exercise benefits/barriers scale (EBBS) [33] and two focus group discussions to explore the participants’ barriers to engaging in PA and the delivery of a planned PA intervention [30].

#### Findings

The results of the study showed that the participants’ barriers to PA were associated with HIV-related symptoms, HIV medications, aging, finances, time-constraints, home environment, a lack of knowledge about the value of PA, and a lack of motivation to engage in PA. In terms of exercise benefits, participants agreed the most with the item: ‘exercise improves the way my body looks’. However, for some benefits, the participants were neutral (e.g., ‘exercise allows me to carry out normal activities without becoming tired’; ‘exercise improves the quality of my work’; and ‘exercise is good entertainment for me’) or tended to ‘agree’ (e.g., ‘my disposition is improved by exercise’). Participants had higher perceived benefits than barriers to exercise. Exercise preferences included aerobics, aerobic exercises, calisthenics and chair exercises. Participants preferred group classes, with monitoring and tracking of exercise.

#### Contributions of the study towards intervention development

Preferred exercises were included in the intervention. The intervention also included educating the participants on the strategies of overcoming the barriers to PA in a low-income neighbourhood. Participants were also taught about the benefits of PA. At the request of the participants, exercise diaries and pedometers were used to monitor PA.

The results of the 4 preliminary studies will be described and highlighted in relation to the APEASE process/BCW framework throughout the results.

Additionally, in order to identify the target behaviours, previous literature was used to draw up a list of possible PA behaviours to be targeted by the intervention [34–38].

### Models and frameworks informing the study

#### Behaviour change wheel

Having noted the need for a comprehensive framework when designing an intervention, Michie et al. [29] reviewed current frameworks and evaluated their effectiveness based upon their comprehensiveness, coherence and association with an overarching behavioural model. Subsequently, a new framework was developed based on an amalgamation of 19 existing frameworks [29]. The BCW links policy to behaviour through various intervention functions. The purpose of the wheel is to aid intervention developers in identifying pertinent potential ingredients needed for behaviour change and, thereby, assist designers in analysing target behaviours and characterising interventions and their active components [29]. The BCW provides clear procedures for linking theory to established behavioural change techniques (BCTs). The ability of the BCW to link theory with BCTs, gives the BCW an advantage over existing frameworks. Other benefits of the BCW are that the model (a) affords one the tools to develop an intervention, even if one does not have a broad knowledge of behaviour change theories, (b) provides a wide choice of intervention features, and (c) offers a division between intervention function and achievement method (policy) [29].

**Fig. 1:**
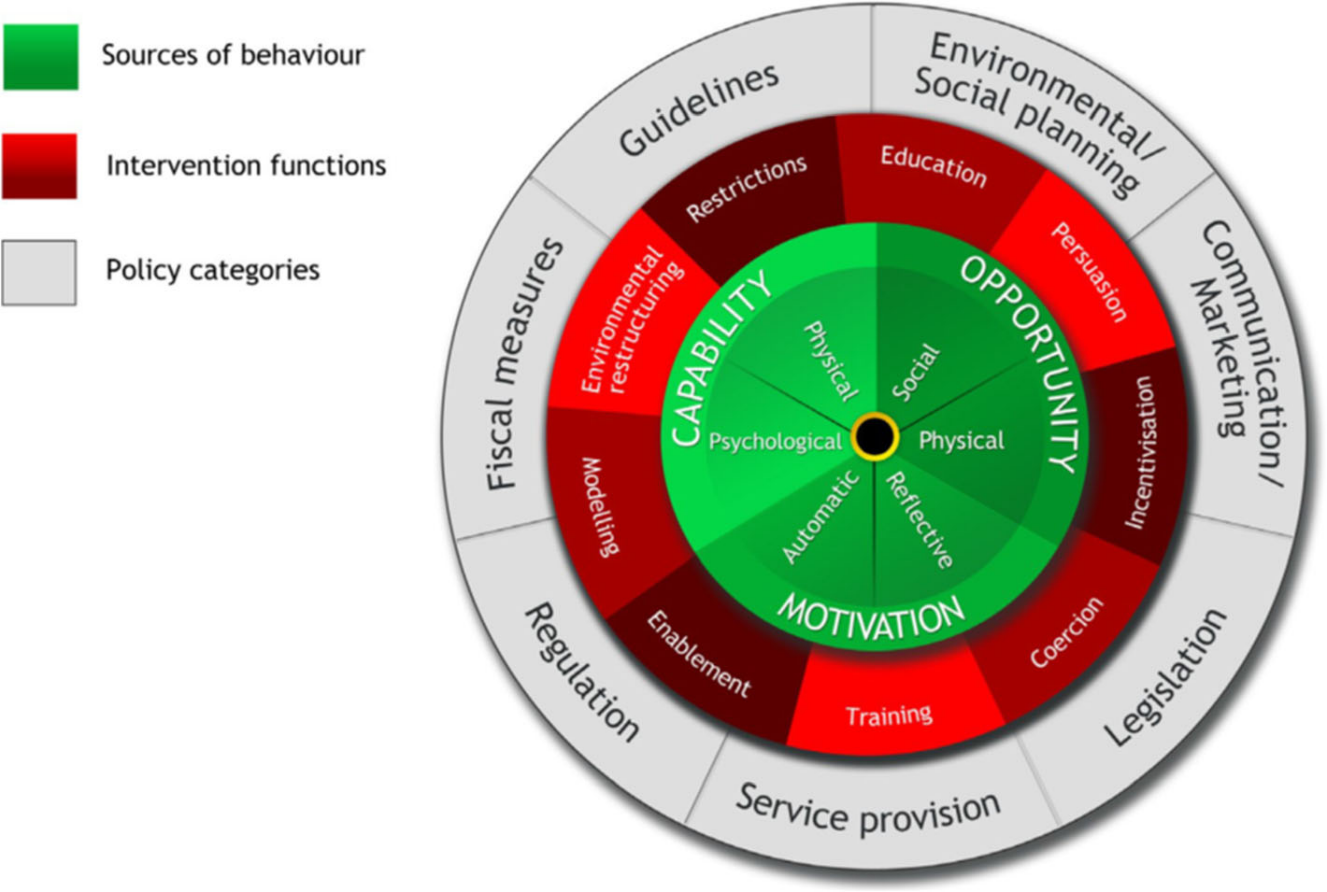
The Behaviour Change Wheel (used with permission from authors) [29]. The behaviour change wheel has three layers, the innermost being the sources of behaviour, namely, capability, opportunity and motivation with each comprising a number of affordances, such as physical, social, etc.; the middle layer comprises nine intervention functions, such as education, persuasion, incentivisation, etc.; and the outermost layer comprises seven policy categories for example, environmental and social planning, communication/marketing, etc. Each of the components within the behaviour change system is not static, but interact with each other in a dynamic manner to explain behaviour change

At the centre of the wheel, are the various sources of behaviour that are necessary for behaviour change to occur (Fig. 1). The BCW utilises a theoretically based behaviour system referred to as the Capability, Opportunity, Motivation–Behaviour (COM-B) model. The model proposes that a change in behaviour will involve a change in at least one of the following components i.e., the capability of a person to carry out that behaviour; or the opportunity for the behaviour to occur; or the motivation to perform the behaviour at that moment in time [29].

Each component of the COM-B model comprises two elements [29]. Capability is composed of physical and psychological abilities, both required to carry out the behaviour. The physical and social environment affords Opportunity, including contextual factors such as time, resources, physical barriers, and social and cultural expectations. Motivation involves use of reflective processing for planning and evaluating a behaviour, and automatic processes triggered by emotion, impulse and reflex reactions [29].

The Theoretical Domains Framework (TDF), also embedded in the BCW, can be used to further understand behaviour. The TDF is made up of 14 domains emanating from theoretical constructs identified from 33 behaviour change theories, and was developed by authorities in behaviour change [39, 40]. The TDF assists intervention designers in choosing and using appropriate theory [41]. The framework is related to the COM-B components, and assists in understanding the target behaviour [29]. The framework includes the following 14 domains, namely, knowledge, skills, memory, attention and decision processes, behavioural regulation, social/professional role and identity, beliefs about capabilities, optimism, beliefs about consequences, intentions, goals, reinforcement, emotion, environmental context and resources, and social influences. The COM-B system and TDF when combined offer a complete theoretical model for understanding behaviour change [42].

The BCW proposes that in order to change behavioural components, an intervention must perform certain functions [29]. The middle layer of the wheel in the BCW represents the intervention functions that are listed in Table 1. The outer layer of the wheel indicates different policies that can be used to apply to interventions. The definitions applied to intervention functions and policies are shown in Table 1.

**Table 1:** Definitions of intervention functions and policies

Intervention functions	Definition
Education	Increasing knowledge or understanding
Persuasion	Using communication to induce positive or negative feelings or stimulate action
Incentivisation	Creating expectation of reward
Coercion	Creating expectation of punishment or cost
Training	Imparting skills
Restriction	Using rules to increase the target behaviour by reducing opportunity to engage in competing behaviours
Environmental restructuring	Changing the physical or social context
Modelling	Providing an example for people to aspire to or imitate
Enablement	Increasing means/reducing barriers to increase capability (beyond education and training) or opportunity (beyond environmental restructuring)
Policies	
Communication/marketing	Using print, electronic, telephonic or broadcast media
Guidelines	Creating documents that recommend or mandate practice. Includes changes to service provision
Fiscal	Using the tax system to reduce or increase the financial cost
Regulation	Establishing rules or principles of behaviour or practice
Legislation	Making or changing laws
Environmental/social planning	Designing/controlling the physical or social environment
Service provision	Delivering a service

Adopted from Michie et al. with permission from authors [29]

#### Use of the behaviour change wheel

The BCW employs eight systematic steps to design an intervention [29]. The first three steps assist intervention designers to identify a specific behaviour they wish to change. Steps one to eight of the BCW are outlined below:

- Step 1: Define the problem to be addressed in behavioural terms.
- Step 2: Select the target behaviour(s), i.e., the behaviours most likely to bring about change to address the problem.
- Step 3: Specify the target behaviour(s) in as much detail as possible.
- Step 4: Identify what component needs to change using the COM-B model.
- Step 5: Select the relevant intervention functions from the following list:education; persuasion; incentivisation; coercion; training; restriction; environmental restructuring; modelling and enablement (based on an assessment of the APEASE criteria: affordability, practicability, effectiveness, acceptability, side effects and safety, and equity) (Table 2).
- Step 6: Select the applicable policy categories to sustain the delivery of the identified intervention functions based on the APEASE criteria.
- Step 7: Select the relevant BCTs based on the APEASE criteria.
- Step 8: Select the mode(s) of intervention delivery based on the APEASE criteria, and confirm the intervention content.

**Table 2:** The APEASE criteria for designing and evaluating interventions

Criterion	Description
Affordability	The ability of the intervention to meet the expense of the intervention. An intervention is affordable if the budget of the intervention allows it to be delivered to, or accessed by, all those for whom it is intended.
Practicability	The viability of the intervention. A practicable intervention is one that can be delivered as designed through the means intended to the target population.
Effectiveness and cost-effectiveness	The effect size of the intervention in relation to the desired objectives in a real world context. Cost-effectiveness refers to the ratio of effect to cost.
Acceptability	The extent to which relevant stakeholders (public, professional and political) judge an intervention appropriate.
Side-effects/Safety	An intervention may be effective and practicable but have unwanted or unintended consequences. This needs to be taken into consideration when planning the intervention.
Equity	The extent to which an intervention may reduce or increase the disparities in standard of living, wellbeing or health between different sectors of society.

Adapted from Michie et al. with permission from authors [29]

### NB: to fully understand the use of the BCW in designing interventions, the steps and the associated tables, the reader is referred to Michie et al. [29]

The BCW was used to implement the evidence from the four preliminary studies [11, 12, 30, 31] into the intervention design process. To design the intervention, the eight steps of the intervention design process using the BCW recommended by Michie et al. where followed [29].

## Results

The results of the study are presented in line with the three stages and the eight steps of the BCW intervention design process, which are recommended by Michie et al. [29].

### The theoretical underpinning of the intervention

The results from the systematic literature review [12] showed that the Transtheoretical model (TTM) of behaviour change and the Social Cognitive theory (SCT) were the common theoretical frameworks used as the basis for most of the successful interventions for individuals of low-SES. Consequently, the planned intervention was informed by the SCT and the TTM as the theoretical underpinnings.

#### Stage 1: understand the behaviour

##### Step 1: defining the problem in behavioural terms

The target population was HIV positive adult women of low SES. The intervention would target women because a preliminary cross-sectional study to inform the intervention design found that PA is significantly higher in men than in women [31]. As such, women were at a higher risk of physical inactivity. The behavioural problem was a lack of regular PA among these individuals. Table 3 shows the questions that the researchers attempted to answer and the answers that were developed by the researchers in order to define the problem in behavioural terms as recommended by Michie et al. [29].

**Table 3:** Defining the problem in behavioural terms

Question	Answer
What behaviour?	Increasing physical activity among PLWHA of low SES.
Where does the behaviour occur?	At the community centre; at home.
Who is involved in performing the behaviour?	HIV positive women of low SES at a community centre.

Adapted from Michie et al. with permission from authors [29]

##### Step 2: selecting the target behaviour

Target behaviours were rated on (a) impact on behaviour change and, (b) likelihood of changing behaviour. The rating of each target behaviour was determined by examining if the target behaviour matched the low-cost target behaviours identified in the systematic review [12]. Studies included in the systematic review only targeted non-costly PA behaviours, such as promoting walking, engaging in PA through activities of daily living [43–45], or alternative exercises, such as chair exercises [46]. Any potential target behaviours that had a measurement score rated as “unpromising” were not included in the intervention. The results of a mixed method study [30] showed that PLWHA preferred aerobics, walking, calisthenics, chair exercises, group exercises and light jogging.

Based on the literature [34–38] and the systematic review [12], the following feasible and low-cost PA behaviours were identified, namely:

1. Walking, e.g., to and from the church, the store etc. [36].
2. Simple home-based exercises, e.g., chair exercises, stepping or stair-climbing, lifting weighted objects, etc. [34].
3. Activities of daily living, e.g., domestic cleaning, sweeping, vacuuming, hanging-up washing, etc. [37].
4. Exercising at the community centre, e.g., organised group exercise classes, either free callisthenic exercises or rhythmical exercises, such as aerobics to music, etc. [38].
5. Exercising during leisure time, e.g., actively participating in age- and health-appropriate activities, such as gardening, knitting, sewing, bowls, darts, table tennis, etc. [35].

Additionally, participants were also supplied with information relating to safety when exercising (e.g., information pertaining to the importance of warm up, stretching, hydration during exercise etc.). Even though questions pertaining to permissible cultural practices among women were not asked, all exercise activities included in the intervention were deemed to be morally, ethically and culturally appropriate.

Table 4 shows how each target behaviour was scored for inclusion using the BCW guide. Leisure-related PA, such as gardening, was also targeted as a target behaviour, because the preliminary research found that PLWHA of low SES engaged less in leisure-related PA [31]. We hoped that the intervention would promote leisure-related PA amongst PLWHA. Walking was also targeted because the same study found that PLWHA of low SES were likely to engage in transport-related PA. All selected PA behaviours were of low to moderate intensity, because the study revealed that PLWHA of low SES were more likely to engage in moderate-intensity PA than vigorous-intensity PA [31].

**Table 4:** Selecting the target behaviours

Potential target behaviours relevant to improving PA engagement among PLWHA of low SES	Impact of behaviour change	Likelihood of changing behaviour	Spill over score	Measurement score
Walking, e.g., to and from church, the store, etc.	*Very promising*	*Very promising*	*Very promising*	Very promising
Simple home-based exercises.	*Very promising*	*Very promising*	*Very promising*	Very promising
Activities of daily living.	*Very promising*	*Very promising*	*Very promising*	Very promising
Exercising at the community centre	*Very promising*	*Very promising*	*Very promising*	Very promising
Exercising during leisure (e.g., actively participating in organised sport)	*Unpromising but worth considering*	*Unpromising*	*Unpromising*	Unpromising

Target behaviours: Walking; Simple home-based exercises; Activities of daily living; exercising at the community centre

Adapted from Michie et al. with permission from authors [29]. Spill over score = score assigned to the target behaviour based on its potential to impact on behaviour change and its likelihood of changing behaviour; Measurement score = Final score assigned to the target behaviour; Very promising = highly likely to change behaviour; Promising = likely to change behaviour; Unpromising, but worth considering = may not change behaviour within the given context but can be considered

##### Step 3: specifying the target behaviour

Michie et al. [29] recommend that when specifying the target behaviour, one has to consider the following questions: Who, What, When, Where, How often and with whom. Table 5 shows the list of the answers generated from answering these questions.

**Table 5:** Specifying the target behaviour

Purpose: To describe the target behaviour according to who needs to do the behaviour*,* what needs to be done, when it needs to be done, where it needs to be done, how often and with whom	
Target behaviours	Walking to-and-from places; Doing simple home-based exercises and engaging in physical activity through activities of daily living; Doing simple exercises at the community centre.
***Who*** needs to perform the behaviour?	PLWHA of low SES
***What*** do they need to do differently to achieve the desired behaviour?	Walk at a moderate-to-vigorous intensity instead of using motorised transport; do home-based exercises; Do simple home-based exercises and activities of daily living at a moderate-to-vigorous intensity; Do simple exercises at the community centre at a moderate-to-vigorous intensity; seek out opportunities for being more active around the home, such as performing domestic chores, e.g., gardening, etc.
***When*** do they need to do it?	When going to-and-from places; during free time at home; during free time at the community centre.
***Where*** do they need to do it?	When going to-and-from places; at home; at the community centre.
***How*** often do they need to do it?	Whenever going to-and-from places; at least once every other weekday for at least 30 min or bouts of 10 min accumulatively.
***With whom*** do they need to do it?	Alone/in a group; alone/ with family and friends; with the exercise leader and other participants attending the community centre.

Adapted from Michie et al. with permission from authors [29]

The intervention would then (What) promote PA among (Who) women of low SES living with HIV and AIDS (Where) at a community centre, and encourage participation (With whom) alone, in a group, or with an exercise leader. Ideally, (How often) group activities will be performed once a week and the participants would be encouraged to engage in PA every day for at least 30 min. In addition, the inclusion of exercises, such as walking, chair exercises, aerobics, calisthenics, aerobic exercises and leisure-related PA in the intervention, where based on the findings from two preliminary studies [30, 31].

##### Step 4: identifying what needs to change

Table 6 shows the outcome of matching participant’s statements obtained through focus group discussions in a preliminary study [30] with the COM-B components in order to identify what needed to change. The findings revealed that physical capability, psychological capability, physical opportunity, social opportunity, and reflective and automatic motivation needed to change for the target behaviour to occur.

**Table 6:** Matching of participant statements in the focus group discussions [30] with the COM-B components to identify what needed to change

*Purpose:* To identify what need*e*d to change for PLWHA of low SES to engage in PA, Using the COM-B model			
COM-B components	What needed to happen for the target behaviour to occur?	Is there a need for change?	Example of participant statement in focus group discussions to support inclusion
*Physical capability*	Developing the physical skills to perform the exercises, such as teaching participants how to correctly do sit-ups.	Yes, change was needed, as most PLWHA of low SES do not possess these skills.	*“I do not know which exercises to do, …*”
*Psychological capability*	Developing knowledge of exercises, the correct techniques used and the appropriate intensity.	Yes, change was needed in terms of knowledge of cost-effective home-based exercises, exercise technique and intensity.	“*…, I do not have the information about exercising”*
*Physical opportunity*	Accessing time, resources and locations to exercise and understanding when an opportunity to engage in PA presents itself.	Yes, participants need*e*d to know the opportune time to exercise, the resources available and when and where to exercise.	*“I simply do not have the time, I come to the community centre every day in the morning, leave late, then at home I have to focus on the domestic chores”*
*Social opportunity*	Increasing social support from family and friends for engaging in PA	Yes, change *was* needed so that PLWHA of low SES can make use of social support to engage in PA	*“Let’s exercise as a group here at the community centre and, then, we can exercise alone at home”*
*Reflective motivation*	Instilling beliefs that engaging in PA will help manage HIV/AIDS by teaching the participants about the importance of PA in managing HIV and HAART-related complications.	Yes, change was needed as most PLWHA of low SES do not know the benefits of PA for managing HIV/AIDS.	*“I thought exercising was for fat individuals, I have already lost weight, due to the disease, so I thought exercising might worsen the situation”*
*Automatic motivation*	Establishing routines and habits for engaging in PA. Automatic motivation would also be derived from weight control, being more energetic, performing activites of daily living (ADLs) a lot easier, and enhanced quality of life (QoL)	Yes, change was needed to establish routines and habits. Change was also needed for compliance and sustainability of the programme.	*“I think we must record our exercise activities somewhere so that we keep track of the exercises we do”*
Behavioural analysis of the relevant COM-B components	Physical capability, psychological capability, physical opportunity, social opportunity, and reflective and automatic motivation need*e*d to change for the target behaviour to occur.		

Adapted from Michie et al. with permission from authors [29]

#### Stage 2: identification of intervention options

##### Step 1: intervention functions

Table 7 shows the outcome of the intervention function selection process based on the APEASE criteria and the studies selected in the literature review [12]. Intervention functions which best met the APEASE criteria (see Table 2) were included in the intervention. The following intervention functions, namely, persuasion, coercion and restriction were excluded, because they did not satisfy the APEASE criteria, while environmental restructuring was not compatible with the intervention theoretical frameworks (see Table 8) identified in the literature review [12]. The selected intervention functions, based on the BCW guide and the systematic literature review, were education, incentivisation, training, modelling and enablement.

**Table 7:** Linking the results of the behavioural assessment with the intervention functions

Candidate intervention functions	Does the intervention function meet the APEASE criteria in the context of engaging PLWHA of low SES in PA?	Application of intervention function based on the studies identified in the systematic literature review [22]
Education	Yes, since it was practicable, as there was time available to teach PLWHA about the benefits of PA; about simple and cheap home-based exercises; about the barriers to PA and how to deal with them; as well as about the social support and self-efficacy for PA.	[43, 46, 48, 50, 52]
Persuasion	Yes, since it was likely to add value to the intervention. However, none of the researchers were trained to apply persuasive messages to motivate participants into adopting PA.	None of the studies
Incentivisation	Yes, since the use of incentives can motivate participants to adhere to the intervention. Food and fruit packs could be used for incentivising.	[49]
Coercion	No, since it does not stimulate the desired autonomous behaviour and can have legislative implications.	None of the studies
Training	Yes, since it was practicable, as there was time to impart exercising skills to participants. Training could be used to teach participants simple home-based exercises or simple exercises to do at the community centre.	[48]
Restriction	No, since it was not practicable, as there was not enough opportunities to apply this intervention function. Resources available did not permit the employment of this function in the intervention.	None of the studies
Environmental restructuring	No, the theoretical frameworks informing the intervention (SCT and TTM) did not allow use of this intervention function (it applies to the Socioecological Model).	[52]
Modelling	Yes, it was practicable to demonstrate how to do the simple home-based exercises and exercises at the community centre.	[46, 48]
Enablement	Yes, it was practicable in order to increase the means for PA participation and reduce the barriers.	[44, 46, 47, 49, 51–53, 67]
SELECTED INTERVENTION FUNCTIONS: Education; incentivisation; training; modelling; enablement		

Adapted from Michie et al. with permission from authors [29]. APEASE = affordability, practicability, effectiveness and cost effectiveness, acceptability, side effects and safety, and equity. NB: Quinn et al. [67] was not identified through the systematic literature review

**Table 8:** Breakdown of specific intervention characteristics as identified in studies included in the literature review by Mabweazara et al. [12]

Study	Specific physical activity behaviours targeted by intervention	Educational component and its focus	Intervention function/s	Policy category	Mode of delivery	Tailoring	Exercise tracking and monitoring
Albright et al. [43]	Teaching participants to accumulate 30 min of moderate-intensity PA e.g. walking 5 days a week.	Used eight 1-h weekly behavioural skill-building classes to inform and motivate women to become more physically active.	Education	Guidelines Service provision	Mailing Phone	Curriculum for the education component was designed to be culturally sensitive for the Latina population.	Pedometers PA self-monitoring logs
Dutton et al. [44]	Taking the stairs; promoting walking; gardening; dancing	Not specified or N/A	Enablement	Service provision	Mailing Phone	Development of individualised goals; Intervention materials were tailored for each participant; recommendations were tailored to the individual’s current activity levels, activity preferences and capabilities.	N/A
Emmons et al. [47]	Not specified or N/A	Taught participants on the social meanings of PA; social support	Enablement	Service provision	Mailing Phone	Intervention takes into account elements of the social context in which participants live.	N/A
Hovell et al. [48]	Not specified or N/A	Included exercise education; proper foot wear; hydration; proper posture; proper attire; importance of warm-up/cool down; exercise safety; injury prevention; treatment of minor injuries; heart-healthy diet and exercise health benefits.	Education Training	Service provision	Face-to-face	Exercise education was culturally appropriate for low-literacy Latinas. Intervention took into consideration participants’ personal concerns.	Heart rate monitors
Keyserling et al. [46]	Chair exercises	Taught problem solving skills; social support; lifestyle behaviour change strategies	Training Education Enablement	Service provision	Mailing Phone Face-to-face	Development of an individualised action plan.	Pedometer
Lowther et al. [49]	Not specified or N/A	Not specified or N/A	Enablement	Service provision	Face-to-face	Not specified or N/A	Not specified or N/A
Marcus et al. [50]	Not specified or N/A	Tip sheets on selected topics e.g. stretching; measuring heart rate	Education Enablement	Service provision	Mailing	Individually tailored feedback reports. The intervention addressed PA barriers identified by Latinas in focus group discussions.	Pedometers PA logs
Pekmezi et al. [51]	Not specified or N/A	Not specified or N/A	Enablement	Service provision	Mailing	PA manuals were matched to participant’s current level of motivational readiness and participants were given individually tailored feedback.	Pedometers PA logs
Pekmezi et al. [45]	Walking	Not specified or N/A	Enablement	Service provision	Mailing Phone	Personalised letters sent to participants; Messages tailored to the participant’s motivational readiness.	N/A
Webel et al. [52]	Was dependent on the outcome of participant’s small self-designed experiments to improve health	Taught systems thinking; self-monitoring and goal setting; lifestyle routines; systems improvement strategies and social support; PA and self-experiments; patterns of exercise, types of fitness and testing small experiments; sleep; mental wellness behaviours; personal time; relapse prevention	Education Enablement	Service provision	Face-to-face	Included challenges specifically faced by PLWH. Individual participants determined and evaluated their own behaviour change. The intervention was culturally adapted.	Diaries of daily activities
Whitehead et al. [53]	Not specified or N/A	Not specified or N/A	Enablement	Service provision	Mailing	Used individually tailored letters	N/A

Participants would have to be trained to be able to perform the home-based exercise activities. Modelling could be used in demonstrating to the participants how to perform the exercise activities. Enablement could also be applied to reduce the barriers to PA by teaching the participants the strategies of overcoming the barriers in order to increase their capability for engaging in regular PA. In addition, incentives could be used to motivate the participants to meet their PA goals. Table 8 shows the intervention characteristics, as identified in studies included in the literature review by Mabweazara et al. [12].

##### Step 2: policy categories

The next step was to identify the relevant policies that would support the delivery of the identified intervention functions. The following policy categories*,* namely*,* communication/marketing, guidelines, regulation, legislation and fiscal measures did not meet the APEASE criteria and were subsequently excluded. The same policy categories were not identified in the systematic review [12]. Service provision was the only policy category that was selected through the APEASE criteria and using the outcomes of the studies included in the systematic review [12]. Table 9 shows the outcomes of selecting the policy category using the APEASE criteria.

**Table 9:** Selecting the policy categories to support the intervention delivery

Intervention function	COM-B component	Potentially useful policy categories	Does the policy category meet the APEASE criteria?
*Education*	Psychological capability; reflective motivation	Communication/marketing	Too expensive, since persons of low income are more likely to be of low literacy levels, as well as being unemployed, so they may not be able to read or access relevant information, including communication technologies.
		Guidelines	No, PA guidelines for PLWHA already exist.
		Regulation	Possibly in the long term, but not at the present time, because rules or principles of practice can only be established over time.
		Legislation	Not practicable in this context, as the intervention does not aim to make or change laws.
		Service provision	Yes, and very practicable, because the intervention designers aim to deliver a service to PLWHA of low SES.
*Incentivisation*	Reflective motivation; automatic motivation	Communication/marketing	Too expensive, since the participants are of low income or unemployed and of low literacy levels and not able to read or afford information communication technologies.
		Guidelines	No, PA guidelines for PLWHA already exist.
		Fiscal measures	Not relevant in this context, this policy uses the tax system to reduce or increase the financial cost in order to change behaviour.
		Regulation	Possible in the long term, but not present moment, because this policy seeks to establish rules or principles of behaviour.
		Legislation	Not practicable in this context, since the intervention does not seek to make or change laws.
		Service provision	Yes, very practicable, because the intervention designers aims to deliver a service to PLWHA of low SES.
*Training*	Physical capability; psychological capability; automatic motivation; physical opportunity	Guidelines	No, PA guidelines for PLWHA already exist.
		Fiscal measures	Not relevant in this context, since this policy uses the tax system to reduce or increase the financial cost in order to change behaviour.
		Regulation	Possible in the long term, but not at the present time, because rules or principles of practice can only be established over time.
		Legislation	Not practicable in this context, since the intervention does not seek to make or change laws
		Service provision	Yes, very practicable, because the intervention designers aim to deliver a service to PLWHA of low SES.
*Modelling*	Psychological capability; reflective motivation; automatic motivation; social opportunity	Communication/marketing	Too expensive, since the participants are of low income or unemployed and of low literacy levels so they may not be able to read or afford information communication technologies
		Service provision	Yes, very practicable, because the intervention designers aim to deliver a service to PLWHA of low SES.
*Enablement*	Psychological capability; reflective motivation; automatic motivation; physical opportunity; social opportunity	Guidelines	No, PA guidelines for PLWHA already exist
		Fiscal measures	Not relevant in this context, since this policy uses the tax system to reduce or increase the financial cost in order to change behaviour.
		Regulation	Possible in the long term, but not at the present time, because rules or principles of practice can only be established over time
		Legislation	Not practicable in this context, since the intervention does not seek to make or change laws.
		Service provision	Yes, very practicable, because the intervention designers aim to deliver a service to PLWHA of low SES.
Policy category selected: Service provision			

Adapted from Michie et al. with permission from authors [29]. APEASE = affordability, practicability, effectiveness and cost effectiveness, acceptability, side effects and safety, and equity

#### Stage 3: identifying intervention content and implementation options

##### Step1: identification of behaviour change techniques

The next step was to select the behaviour change techniques (BCTs) that were most likely to be suitable for inclusion in an intervention to engage PLWH in PA. For the definitions of the different BCTs, the reader is referred to Michie et al. [29]. Table 10 presents the selected BCTs from the BCW and those identified through the systematic literature review [12].

**Table 10:** Matching of BCTs identified in the systematic literature review to those identified through the BCW

BCTs identified in the systematic review	BCTs identified through the BCW
Provide information on the consequences of the behaviour to the individual	Information about health consequences
Provide information on the consequences of the behaviour in general	
Provide feedback on the performance	Feedback on the behaviour
Prompt review of the outcome goals	Feedback on outcomes of the behaviour Goal-setting outcome
Prompt self-monitoring of the behavioural outcomes	Self-monitoring of the behaviour
Prompt self-monitoring of the behaviour	Self-monitoring of the behaviour
Model/Demonstrate the behaviour	Demonstration of the behaviour
Provide instruction on how to perform the behaviour	Instruction on how to perform the behaviour
Plan social support/social change	Social support (unspecified) Social support (practical)
Goal-setting behaviour	Goal-setting behaviour
Prompt review of the behavioural goals	Review the behavioural goals
Action planning	Action planning

Selected BCTs: Information about health consequences; feedback on behaviour; feedback on outcomes of the behaviour; goal setting outcome; self-monitoring of the behaviour; demonstration of the behaviour; instruction on how to perform the behaviour; social support (unspecified); social support (practical); goal setting behaviour; review behavioural goals; action planning

##### Step 2: mode of delivery

Most of the studies in the systematic literature review [12] used mailing and the telephone as a method of delivery. However, the low SES of the participants in the current planned intervention would not allow for mailing or use of the telephone as feasible modes of delivery. A face-to-face mode of delivery was, thus, deemed most appropriate for the current intervention.

Selection of the face-to-face mode of delivery was also supported by evidence from the studies in the systematic literature review [12]. For example, Hovell et al. [48], Keyserling et al. [46] and Webel et al. [52] used the face-to-face method successfully. Table 11 presents the outcome of the APEASE criteria used to select the intervention mode of delivery.

**Table 11:** Outcome of the APEASE criteria for selecting the mode of delivery

Mode of delivery				Does the mode of delivery meet the APEASE criteria in the context of increasing PA for PLWHA of low SES?
Face-to-face	Individual			Yes
	Group			Yes
Distance	Population level	Broadcast media	TV	These modes of delivery are not relevant, as PLWHA of low SES are unlikely to have access to phones, computers or be exposed to other forms of media.
			Radio	
		Outdoor media	Billboard	
			Poster	
		Print media	Newspaper	
			Leaflet	
		Digital media	Internet	
			Mobile phone application	
	Individual level	Phone	Phone helpline	
			Mobile phone text	
		Individually accessed computer programme		

Adapted from Michie et al. with permission from authors [29]. *APEASE* affordability, practicability, effectiveness and cost effectiveness, acceptability, side effects and safety, and equity

## Discussion

This study described the preliminary and developmental research to identify key components of a contextualised intervention for promoting PA among PLWHA of low SES. The BCW has been used previously in the development of behavioural change interventions [54–56]. The current study specifically focused on the development of a contextualised PA intervention for PLWHA of low SES. PLWHA of low SES are a high risk group for inactivity [9, 10]. To the best of our knowledge, no study has been conducted to develop a PA intervention for PLWHA of low SES using the BCW, especially in an African context.

The outcome: A contextualized physical activity intervention for people living with HIV and AIDS of low socioeconomic status.

This section of the paper describes the contextualised PA intervention for PLWHA which was developed using strategies outlined in this paper. The intervention was conducted at a community centre caring for HIV positive Black African Xhosa-speaking women in a low-income community in the Western Cape Province in South Africa. The efficacy of the PA intervention was tested through a six-week randomised cross-over trial and participants exposed to the PA intervention had significant increases in PA compared to those in the standard care group [57]. The PA intervention is referred to as contextualised, because it employs a combination of strategies and information tailored for HIV positive women of low SES and considers the participants’ preferred content (exercise activities, barriers to PA, monitoring and tracking of PA). The intervention was informed by the SCT [58] and the TTM [59].

The intervention was delivered in the indigenous isiXhosa language, the vernacular of the participants. Fruit packs and food packs were used for incentivising the participants at each training session to promote adherence. The intervention employed strategies that highlighted low-cost, local PA resources and opportunities to help HIV infected women of low SES to engage in PA. The intervention emphasised increasing walking, doing simple home-based exercise activities, such as sit-ups and lunges, as well as engaging in activities of daily living (ADLs), such as gardening and washing clothes manually.

The intervention started by determining each participant’s stage of readiness to engage in PA. In addition, specific and measurable short-term goals were set with each participant. Short term-goals were those which could be achieved in six days (i.e.) before our next contact with the participants. Participants were also shown how to use the pedometer and the PA diary. Participants then attended PA classes for two hours each week over a period of six weeks, where they were taught the following topics in the first hour, namely: (a) the role of PA as a non-pharmacological, low-cost strategy for the management of HIV, (b) the health benefits of PA for PLWHA, (c) safety considerations when exercising, (d) how to identify or develop simple home-based exercises, such as chair exercises, (f) strategies for increasing physical exercise self-efficacy and social support, and (g) how to identify the barriers to PA and strategies for overcoming them. These classes were held at a community centre caring for HIV positive Black African Xhosa-speaking women of low SES in Nyanga Township in the Western Cape Province in South Africa. In the second hour, participants were taught and performed home-based exercise activities for 30 min and, then, a 30-min group aerobics exercise session was done. Participants received a PA manual with all the material taught during the classes. All exercise activities were in line with the American College of Sport Medicine (ACSM) guidelines for individuals infected with HIV [60].

Goals were set on the number of steps and the number of home-based exercises to do per day that were tailored to the participants’ needs and abilities. Individualised feedback followed on a weekly basis and goals were reviewed and changed accordingly. Feedback to participants corresponded to their current motivational readiness for PA. In the last week of the intervention, the participant with the highest number of steps recorded on the pedometer received an achievement award.

Participants were also educated on the barriers to PA and how to overcome them. Participants identified their perceived barriers to PA and then discussions followed on how to overcome them. Strategies to overcome barriers and increase PA included providing information on where and when to exercise. Participants were also taught how to manage their time for PA, e.g., if a participant mentioned that they did not have adequate time for PA, they were taught to integrate PA into their activities of daily living, such as brisk walking to the shop.

Our findings, through the use of the BCW revealed that a contextualised PA intervention for PLWHA of low SES would include low-cost PA activities such as ADLs and walking. Contrastingly, other researchers focusing on the development and contextualisation of PA interventions for individuals of low SES have reported that, low-cost interventions that include walking tend to be taken up by educated, white, middle aged women [61]. Other similar low-cost interventions for persons of low SES, that include walking, have been found to be fraught with barriers, to an extent that participants can’t possibly benefit from them [62]. For example, participants of low SES are reported to view walking as being of little purpose with little understanding of the health benefits of walking [63]. As such, researchers are encouraged to include educational sessions in their interventions for participants to understand the health benefits of different types of physical activities.

PA may be an effective alternative therapy to address the varying health challenges faced by PLWHA, which include the common prevalence of cardiovascular disease, metabolic syndrome, and neurocognitive deficits that are mainly caused by antiretroviral therapy [64]. The use of cost-effective home based exercise activities and integrating PA into activities of daily living in PA interventions for PLWHA of low SES may assist in promoting PA among this population. Costly PA interventions that include high-intensity PA may not work for PLWHA [12, 65].

Additionally, the use of preliminary research may aid in informing the intervention development process. This may be particularly so, if the research is conducted among people who exhibit the same characteristics (e.g., chronic disease and low SES), as those to be targeted by the intervention. For example, in one of our preliminary studies we found that women living with HIV and AIDS prefer exercising as a group with monitoring and tracking of exercise [30]. As such, the final PA intervention included group exercises and the use of pedometers and PA diaries to track and monitor PA.

### Strengths of the study

The intervention was developed by applying a rigorous systematic method that combined theory, participant preferences and practical considerations using the combined evidence from the accumulation of four previous studies. Two theoretical frameworks, the SCT and the TTM, also informed the intervention. The strength of the BCW is that the framework was developed from theoretical constructs of numerous theories, instead of one theory [29]. Multiple theories allow for the use of different strategies or BCTs thereby offering greater opportunity for inducing change [66]. In addition, the breadth of the BCW offers a comprehensive way of tackling possible obstacles and understanding enablers of behaviour change [40].

### Limitations of the study

Even though the BCW is a comprehensive framework for intervention development, subjectivity is still an issue in the choice of intervention content and delivery options [40]. For example, in terms of intervention functions and policy categories, intervention choices depended on the decisions of the intervention developers (SZM; LLL; CL).

## Conclusion

PA can be used to ameliorate the adverse effects of HIV infection and those of antiretroviral therapy among PLWHA. PLWHA of low SES encounter multiple factors that hinder their ability to engage in healthy levels of PA. Cost-effective and contextualised PA interventions are ideal for PLWHA of low SES, and have the potential of promoting regular PA. The BCW framework can be used to design a cost-effective and contextualised PA intervention for PLWHA of low SES. The BCW is a useful and comprehensive framework for intervention design.

## Data Availability

Not applicable.

## Abbreviations

AIDS: Acquired Immunodeficiency Syndrome
BCT: Behaviour change technique
BCW: Behaviour Change Wheel
BMI: Body mass index
COM-B: Capability, opportunity, motivation, behaviour
HIV: Human immunodeficiency virus
PLWA: People living with HIV and AIDS
SCT: Social cognitive theory
TDF: Theoretical Domains Framework
TTM: Transtheoretical Model
WHR: Waist-to-hip ratio

## References

[1] Eijkemans M, Mommers M, Jos MT, Thijs C, Prins MH (2012). Physical activity and asthma: a systematic review and meta-analysis. PLoS One.

[2] Holtermann A, Marott JL, Gyntelberg F, Søgaard K, Suadicani P, Mortensen OS, Prescott E, Schnohr P (2013). Does the benefit on survival from leisure time physical activity depend on physical activity at work? A prospective cohort study. PLoS One.

[3] Lundgren JD, Battegay M, Behrens G, De Wit S, Guaraldi G, Katlama C, Martinez E, Nair D, Powderly WG, Reiss P, Sutinen J (2008). European AIDS clinical society (EACS) guidelines on the prevention and management of metabolic diseases in HIV. HIV Med.

[4] Jaggers JR, Hand GA (2016). Health benefits of exercise for people living with HIV: a review of the literature. Am J Lifestyle Med.

[5] Vancampfort D, Mugisha J, Rosenbaum S, Firth J, De Hert M, Probst M, Stubbs B (2016). Cardiorespiratory fitness levels and moderators in people with HIV: a systematic review and meta-analysis. Prev Med.

[6] Somarriba G, Neri D, Schaefer N, Miller TL (2010). The effect of aging, nutrition, and exercise during HIV infection. HIV AIDS (Auckl).

[7] da Silva Paes L, Borges JP, dos Santos FM, de Oliveira TP, Dupin JG, Harris EA, Farinatti P (2015). Effects of a 2-year supervised exercise program upon the body composition and muscular performance of HIV-infected patients. Open AIDS J.

[8] Tanaka LF, de Oliveira MD, da Silva AM, de Oliveira Konstantyner TC, Peres SV, de Sousa Marques HH (2015). High prevalence of physical inactivity among adolescents living with HIV/AIDS. Revista Paulista de Pediatria (English Edition).

[9] Smit E, Crespo CJ, Semba RD, Jaworowicz D, Vlahov D, Ricketts EP, Ramirez-Marrero FA, Tang AM (2006). Physical activity in a cohort of HIV-positive and HIV-negative injection drug users. AIDS Care.

[10] Murenzi A. Physical activity levels among people living with HIV/AIDS treated with high active antiretroviral therapy in Rwanda. 2011. Available from http: //etd.uwc.ac.za/xmlui/handle/11394/1563. Accessed 20 May 2015.

[11] Mabweazara SZ, Leach LL, Ley C, Smith M, Jekauc D, Dave J, Levitt N, Lambert VE. Physical activity behaviours of persons living with HIV of low socioeconomic status: domain, intensity and sociodemographic correlates. AIDS Care. 2018:1–5. 10.1080/09540121.2018.1493184 29962226

[12] Mabweazara SZ, Leach LL, Ley C (2016). Physical activity interventions for the management of chronic disease in low-income populations: a systematic review. AJPHES..

[13] Muronya W, Sanga E, Talama G, Kumwenda JJ, van Oosterhout JJ (2011). Cardiovascular risk factors in adult Malawians on long-term antiretroviral therapy. Trans R Soc Med Hyg.

[14] Edward AO, Oladayo AA, Omolola AS, Adetiloye AA, Adedayo PA (2013). Prevalence of traditional cardiovascular risk factors and evaluation of cardiovascular risk using three risk equations in Nigerians living with human immunodeficiency virus. N Am J Med Sci.

[15] Olsen MF, Kæstel P, Tesfaye M, Abdissa A, Yilma D, Girma T, Mølgaard C, Faurholt-Jepsen D, Christensen DL, Brage S, Andersen ÅB (2015). Physical activity and capacity at initiation of antiretroviral treatment in HIV patients in Ethiopia. Epidemiol Infect.

[16] Chopra M, Ford N (2005). Scaling up health promotion interventions in the era of HIV/AIDS: challenges for a rights based approach. Health Promot Int.

[17] Myburgh KH, De Bruto PC (2008). Body composition in women with HIV/AIDS: the relevance of exercise. CME: Your SA Journal of CPD.

[18] Ogunmola OJ, Oladosu YO, Olamoyegun MA (2014). Relationship between socioeconomic status and HIV infection in a rural tertiary health center. HIV AIDS (Auckl)..

[19] Kasirye Ibrahim (2016). HIV/AIDS Sero-prevalence and Socio-economic Status: Evidence from Uganda. African Development Review.

[20] Brennan Laura K., Brownson Ross C., Hovmand Peter (2012). Evaluation of Active Living by Design. American Journal of Preventive Medicine.

[21] UNAIDS. HIV Prevalence [Data Sheet]. No date [cited 2018 Nov 10]. Available from: aidsinfo.unaids.org

[22] Statistics South Africa. Statistical release P0302: Mid-year population estimates 2017. Statistics South Africa, Pretoria. [updated 2018 July 23; cited 2018 Nov 10]. Available from: http://www.statssa.gov.za/publications/P0302/P03022018.pdf

[23] Gaskin Cadeyrn, Orellana Liliana (2018). Factors Associated with Physical Activity and Sedentary Behavior in Older Adults from Six Low- and Middle-Income Countries. International Journal of Environmental Research and Public Health.

[24] Allender S, Foster C, Boxer A (2008). Occupational and nonoccupational physical activity and the social determinants of physical activity: results from the health survey for England. J Phys Act Health.

[25] Greaves CJ, Sheppard KE, Abraham C, Hardeman W, Roden M, Evans PH, Schwarz P (2011). Systematic review of reviews of intervention components associated with increased effectiveness in dietary and physical activity interventions. BMC Public Health.

[26] Barker F, Atkins L, de Lusignan S (2016). Applying the COM-B behaviour model and behaviour change wheel to develop an intervention to improve hearing-aid use in adult auditory rehabilitation. Int J Audiol.

[27] Fillipas S, Cicuttini FM, Holland AE, Cherry CL (2013). Physical activity participation and cardiovascular fitness in people living with human immunodeficiency virus: a one-year longitudinal study. J AIDS Clinic Res S.

[28] Knackfuss MI, Maia MC, Soares TCM. Psychological variables and adherence to physical activity programs. In: Watson RR, editor. Health of HIV infected people: food, nutrition and lifestyle with antiretroviral drugs; 2015. p. 511–522. Available from https://books.google.co.za/books?hl=en&lr=&id=N-ScBAAAQBAJ&oi=fnd&pg=PP1&dq=Maria+Irany+Knackfuss+Ubilina+Maria+da+&ots=aZIoxI_Cdo&sig=CPL3bRaRwy4Kp-GR6spBFy6Zf_s#v=onepage&q=Maria%20Irany%20Knackfuss%2C%20Ubilina%20Maria%20da&f=false. Accessed 21 November 2017.

[29] Michie S, Atkins L, West R (2014). The behaviour change wheel: a guide to designing interventions.

[30] Mabweazara SZ, Leach LL, Ley C (2017). Physical activity among HIV positive women of low socioeconomic status: benefits and barriers. AJPHES..

[31] Mabweazara SZ, Leach LL, Ley C (2018). Physical, demographic and socioeconomic predictors of physical activity among people living with HIV of low socioeconomic status.

[32] Deeks SG, Lewin SR, Havlir DV (2013). The end of AIDS: HIV infection as a chronic disease. Lancet..

[33] Sechrist KR, Walker SN, Pender NJ (1987). Development and psychometric evaluation of the exercise benefits/barriers scale. Res Nurs Health.

[34] Atienza Audie A. (2001). Home-Based Physical Activity Programs for Middle-Aged and Older Adults: Summary of Empirical Research. Journal of Aging and Physical Activity.

[35] Pate RR, Ward DS, Saunders RP, Felton G, Dishman RK, Dowda M (2005). Promotion of physical activity among high-school girls: a randomized controlled trial. Am J Public Health.

[36] Matthews CE, Wilcox S, Hanby CL, Der Ananian C, Heiney SP, Gebretsadik T, Shintani A (2007). Evaluation of a 12-week home-based walking intervention for breast cancer survivors. Support Care Cancer.

[37] Opdenacker J, Boen F, Coorevits N, Delecluse C (2008). Effectiveness of a lifestyle intervention and a structured exercise intervention in older adults. Prev Med.

[38] Beedie C, Mann S, Jimenez A (2014). Community fitness center-based physical activity interventions: a brief review. Curr Sports Med Rep.

[39] Michie S (2005). Making psychological theory useful for implementing evidence based practice: a consensus approach. Quality and Safety in Health Care.

[40] Cane J, O’Connor D, Michie S (2012). Validation of the theoretical domains framework for use in behaviour change and implementation research. Implement Sci.

[41] Atkins L, Francis J, Islam R, O’Connor D, Patey A, Ivers N, Foy R, Duncan EM, Colquhoun H, Grimshaw JM, Lawton R (2017). A guide to using the theoretical domains framework of behaviour change to investigate implementation problems. Implement Sci.

[42] Salmon VE. Development of a physical activity intervention for managing fatigue in rheumatoid arthritis (doctoral dissertation). 2016. Available from: http://eprints.uwe.ac.uk/25760/9/160201%20thesis%20FINAL.pdf. Accessed 20 May 2015.

[43] Albright CL, Pruitt L, Castro C, Gonzalez A, Woo S, King AC (2005). Modifying physical activity in a multiethnic sample of low-income women: one-year results from the IMPACT (increasing motivation for physical ACTivity) project. Ann Behav Med.

[44] Dutton GR, Martin PD, Welsch MA, Brantley PJ (2007). Promoting physical activity for low-income minority women in primary care. Am J Health Behav.

[45] Pekmezi DW, Barbera BL, Bodenlos JS, Jones GN, Brantley PJ (2009). Promoting physical activity in low income African Americans: project LAPS. J Health Dispar Res Pract.

[46] Keyserling TC, Hodge CD, Jilcott SB, Johnston LF, Garcia BA, Gizlice Z, Gross MD, Saviñon CE, Bangdiwala SI, Will JC, Farris RP (2008). Randomized trial of a clinic-based, community-supported, lifestyle intervention to improve physical activity and diet: the North Carolina enhanced WISEWOMAN project. Prev Med.

[47] Emmons KM, Stoddard AM, Fletcher R, Gutheil C, Suarez EG, Lobb R, Weeks J, Bigby JA (2005). Cancer prevention among working class, multiethnic adults: results of the healthy directions–health centers study. Am J Public Health.

[48] Hovell MF, Mulvihill MM, Buono MJ, Liles S, Schade DH, Washington TA, Manzano R, Sallis JF (2008). Culturally tailored aerobic exercise intervention for low-income Latinas. Am J Health Promot.

[49] Lowther M, Mutrie N, Scott EM (2002). Promoting physical activity in a socially and economically deprived community: a 12 month randomized control trial of fitness assessment and exercise consultation. J Sports Sci.

[50] Marcus BH, Dunsiger SI, Pekmezi DW, Larsen BA, Bock BC, Gans KM, Marquez B, Morrow KM, Tilkemeier P (2013). The Seamos Saludables study: a randomized controlled physical activity trial of Latinas. Am J Prev Med.

[51] Pekmezi DW, Neighbors CJ, Lee CS, Gans KM, Bock BC, Morrow KM, Marquez B, Dunsiger S, Marcus BH (2009). A culturally adapted physical activity intervention for Latinas: a randomized controlled trial. Am J Prev Med.

[52] Webel AR, Moore SM, Hanson JE, Salata RA. The rationale, design, and initial efficacy of SystemCHANGE™-HIV: a systems-based intervention to improve physical activity in people living with HIV. J AIDS Clin Res. 2013;4(3). 10.4172/2155-6113.1000200 PMC3875215 24383041

[53] Whitehead D, Bodenlos JS, Cowles ML, Jones GN, Brantley PJ (2007). A stage-targeted physical activity intervention among a predominantly African-American low-income primary care population. Am J Health Promot.

[54] Webb J, Foster J, Poulter E (2016). Increasing the frequency of physical activity very brief advice for cancer patients. Development of an intervention using the behaviour change wheel. Public Health.

[55] Connell LA, McMahon NE, Redfern J, Watkins CL, Eng JJ (2015). Development of a behaviour change intervention to increase upper limb exercise in stroke rehabilitation. Implement Sci.

[56] Gould GS, Bar-Zeev Y, Bovill M, Atkins L, Gruppetta M, Clarke MJ, Bonevski B (2017). Designing an implementation intervention with the behaviour change wheel for health provider smoking cessation care for Australian indigenous pregnant women. Implement Sci.

[57] Mabweazara SZ, Leach LL, Ley C, Smith M (2018). A six week contextualised physical activity intervention for women living with HIV and AIDS of low socioeconomic status: a pilot study. AIDS Care.

[58] Bandura A. Social foundations of thought and action: a social cognitive theory. Englewood cliffs, N.J: Prentice-Hall; 1986.

[59] Prochaska JO, Velicer WF (1997). The transtheoretical model of health behavior change. Am J Health Promot.

[60] American College of Sports Medicine. ACSM's guidelines for exercise testing and prescription. Nineth edition. Philadelphia: Lippincott Williams & Wilkins; 2014.

[61] Foster CE, Brennan G, Matthews A, McAdam C, Fitzsimons C, Mutrie N (2011). Recruiting participants to walking intervention studies: a systematic review. Int J Behav Nutr Phys Act.

[62] Hanson S, Guell C, Jones A (2016). Walking groups in socioeconomically deprived communities: a qualitative study using photo elicitation. Health Place.

[63] Hanson S, Cross J, Jones A (2016). Promoting physical activity interventions in communities with poor health and socio-economic profiles: a process evaluation of the implementation of a new walking group scheme. Soc Sci Med.

[64] Yahiaoui A, McGough EL, Voss JG (2012). Development of evidence-based exercise recommendations for older HIV-infected patients. J Assoc Nurses AIDS Care.

[65] Montoya JL, Wing D, Knight A, Moore DJ, Henry BL (2015). Development of an mHealth intervention (iSTEP) to promote physical activity among people living with HIV. J Int Assoc Provid AIDS Care.

[66] Webb Thomas L, Joseph Judith, Yardley Lucy, Michie Susan (2010). Using the Internet to Promote Health Behavior Change: A Systematic Review and Meta-analysis of the Impact of Theoretical Basis, Use of Behavior Change Techniques, and Mode of Delivery on Efficacy. Journal of Medical Internet Research.

[67] Quinn L, Trubey R, Gobat N, Dawes H, Edwards RT, Jones C, Townson J, Drew C, Kelson M, Poile V, Rosser A (2016). Development and delivery of a physical activity intervention for people with Huntington disease: facilitating translation to clinical practice. J Neurol Phys Ther.

